# Psychometric evaluation of the Connor-Davidson Resilience Scale among Iranian population

**DOI:** 10.1186/s12888-023-04580-8

**Published:** 2023-02-07

**Authors:** Hamid Sharif Nia, Long She, Erika Sivarajan Froelicher, João Marôco, Mozhgan Moshtagh, Sima Hejazi

**Affiliations:** 1 Traditional and Complementary Medicine Research Center, Addiction Institute, Mazandaran University of Medical Sciences, Sari, Iran; 2 Faculty of Business, Design and Arts, Swinburne University of Technology, Sarawak, Malaysia; 3 Department of Physiological Nursing, Schools of Nursing, Department of Epidemiology and Biostatistics, School of Medicine, University of California San Francisco, San Francisco, CA 94143-0610 USA; 4 William James Centre for Research. ISPA - Instituto Universitário, Lisbon, Portugal; 5 Faculty of Health, Social Determinants of Health Research Centre, Birjand University of Medical Sciences, Ghaffari Street, Birjand, Iran; 6 Department of Nursing, Bojnurd Faculty of Nursing, North Khorasan University of Medical Sciences, Shahriar Street, Bojnurd, North Khorasan Iran

**Keywords:** Psychological resilience, Questionnaire design, Connor-Davidson Resilience Scale, Psychometric, Iran

## Abstract

**Background:**

The resilience construct is considered a personal trait composed of multiple aspects. Connor–Davidson Resilience Scale is a standard tool composed of five factors and 25 items. This study aimed to determine the psychometric properties of this scale.

**Methods:**

In this cross-sectional study, after the scale translation, the factorial structural validity was assessed via the confirmatory factor analysis with 70 180 samples. Internal consistency, composite reliability, convergent validity were assessed by calculating Cronbach’s alpha, composite reliability, maximum reliability, and Average Variance Extracted. The discriminant validity was assessed using Heterotrait-monotrait ratio of correlations matrix and also, measure invariance was evaluated.

**Results:**

The original five-factor model had good model fit indices but due to low factor loading of item 2 and 20, the model was modified. The Cronbach’s alpha and composite reliability for four factors were above 0.7 (except for factor 5). The convergent validity for all five factors were achieved. Between factors 1 with 2 and 4, 2 with 3 and 4 discriminant validity was not established (correlations > 0.9) and the results suggested that there might be a second-order latent construct behind these factors. Therefore, a second-order assessment was performed. The results of the second-order latent construct assessment showed a good goodness-of fit and strong measurement invariance for both men and women.

**Conclusion:**

The 23-item version of Connor-Davidson Resilience Scale is a reliable and valid scale to measure resilience as a complex construct in the Iran context.

## Introduction

Stress is an unavoidable part of life in the modern era, and according to estimates, the prevalence of traumatic events is nearly 71% in the life course [1]. Therefore adapting to challenging circumstances and crises is essential for the well-being of the general population as part of human development [2]. The resilience construct is recognized as having a significant potential benefit for health promotion in the life course and is a considerable indicator in developing research, policies, and practices on mental health [3].

Some researchers have conceptualized resilience as an individual trait or capacity, but others consider it as an outcome or dynamic process [4]. Despite available definitions, all researchers aim to understand how people could persist against pressures or respond efficiently to trauma and shocking events without having a harmful impacts on health; or even achieving inner growth and thrive [5, 6]. The Coronavirus disease of 2019 (COVID-19) pandemic was a traumatic event that had extreme drawbacks that affected the general population worldwide [7, 8]. Being under severe stress due to an unpredictable disease, disruption in living conditions, healthcare services and communications, and fear of loss and damages (life and economic issues) were all severe threats to different populations, especially for those with limited resources [7].

One strategy to protect mental health globally against this crisis was focusing on individual’s strengths, such as resilience to increase their endurance against adversities [3]. Measuring the resilience in such situations is order valuable in to recognizing individuals at risk, as mental problems could be prevented or reversed through effective interventions and policies [9].

However, finding a standard measure is difficult due to the complexity of characteristics constituting the resilience construct among diverse populations in a different cultures like Iran [10–12]. The Connor-Davidson Resiliency Scale (CD-RISC) identifies a comprehensive and valid measure of resiliency both for the general population and clinical groups in different populations and languages [6, 13, 14].

CD-RISC is a standard tool composed of five factors and 25 items, that is used most often [9]. Based on CD-RISC, the resilience construct is considered a personal trait composed of multiple aspects, including competence, tolerance, accepting or adapting to change, confidence in relationships, power to control, spiritual strength, and thriving after stress) [15].

Therefore, our study question is, does the CD-RISC 25 have acceptable reliability and validity in the general Iranian population during COVID-19?

## Methods

### Design and participants

This study used a cross-sectional design. The online survey was conducted in March 2020 through an Iranian survey platform named Uniform Resource Locator (URL). The survey was distributed via social media and Iranian population were invited to complete the online questionnaire using a convenience sampling. The independent t-test and one-way analysis of variance (ANOVA) test were used to compare the resilience score in different groups based on demographic characteristics.

### Translation

After obtaining written permission from the developer of the scale, according to the guideline proposed by Beaton et al. [16] the CD-RISC was translated to Farsi. The two independent translators translated the CD-RISC into a Farsi version. Afterwards, these two Farsi versions of the CD-RISC were assessed by a group of experts, to develop a single Farsi version of the CD-RISC. Finally, the single Farsi version of CD-RISC was back-translated to English by a Farsi–English translator. The same group of experts confirmed the final version of the scale.

### Factorial structural validity

This study conducted confirmatory factor analysis (CFA) to identify the factorial structure as well as the construct validity and reliability of the Farsi version of CD-RISC using AMOS version 27. The CFA was conducted using maximum likelihood. The model fit was assessed through a number of fit indices, such as Chi-square (*χ*^2^) test, *χ*^2^/degree of freedom(*df*) ratio < 4, comparative fit index (CFI) > 0.90, normed fit index (NFI) > 0.90, and Tucker–Lewis index (TLI) > 0.90, standardized root mean square residual (SRMR) < 0.09, and root mean square error of approximation (RMSEA) < 0.08 [17–19].

### Construct validity and reliability

The internal consistency (Cronbach’s alpha), composite reliability (CR), and maximum reliability (MaxR), and Average Variance Extracted (AVE) were assessed. To achieve acceptable construct reliability, Cronbach’s alpha, CR, and MaxR should be greater than 0.7. For convergent validity, CR should be higher than 0.7, and AVE should be greater than 0.5 [17, 18]. Moreover, the discriminant validity was assessed using Heterotrait-monotrait ratio of correlations (HTMT) matrix, and all values in the HTMT matrix should be less than 0.85 to achieve discriminat validity [20].

### Multivariate normality and outliers

The univariate distributions were tested for outliers, skewness, and kurtosis. The normality of the multivariate distribution was assessed using Mardia’s coefficient of multivariate kurtosis, and the Mardia’s coefficient (> 7.98). Moreover, the outliers of the multivariate distribution were detected using Mahalanobis distance (*p* < 0.001) [21].

### Measurement invariance for gender

Measurement invariance was analyzed by comparing a set of increasing constrained models for men and women: from no constraint to factor loading and intercepts (configural invariance), constrained factor loadings for men and women (metric or weak invariance), and constrained factor loadings and intercepts for men and women (scalar or strong invariance). The Δχ^2^, ΔCFI and ΔRMSEA from two consecutive models were used for evidence of invariance when Δχ^2^ was not significant and/or ΔCFI and ΔRMSEA were less than 0.01 [22].

## Results

### Demographic

A total of 70 180 Iranian individuals completed the online questionnaire. As shown in Table 1, the sample of this study consisted of 25 037 (35.7%) men and 45 143 women (64.3%) with a mean age of 41.24 (SD = 11.71) years. Older adults had higher levels of resilience than younger adults and these differences were significant (*p* < 0.001). Also, there was a statistically difference between men and women’s mean score of resilience (*p* < 0.001). Most participants were married (75.8%) and had a moderate or poor economic status (93.7%). Married individuals were also those with moderate to poor economic status, and they had higher mean score in resilience and they were statistically significant (*p* < 0.001). With respect to the level of education, 63.4% of the total respondents held a bachelor’s degree. Individuals with higher education had higher level of resilience and these differences were also statistically significant (*p* < 0.001). As for the disease history, 80.9% of the participants reported no disease history. The details of the participants’ demographic profile are shown in Table 1.

**Table 1:** Characteristics of participants according to the demographic variables and resilience (*n* = 70 180)

Variables	n (%)	Resilience Mean (SD)	*p*- value
**Gender**			
Male	25 037 (35.7)	62.03 (14.93)	t: 27.369, df: 70 178 *p*< 0.001^*^
Female	45 143 (64.3)	58.67 (15.93)	
**Age (years)**			
< 30	11 586 (16.5)	58.27 (15.85)	F(3, 68,591): 129.176 *p*< 0.001^**^
31–40	24 513 (35.7)	59.04 (15.87)	
41–50	17 728 (25.8)	60.48 (15.48)	
51–99	14 786 (21.6)	61.55 (15.17)	
**Marital status**			
Single	16 998 (24.2)	58.62 (16.23)	t:-10.791, df: 70 178 *p*< 0.001^*^
Married	53 182 (75.8)	60.19 (15.45)	
**Chronic pre-existing conditions**			
No	56 778 (80.9)	59.82 (15.65)	t: -1.904, df: 20 137.104 *p*> 0.05^*^
yes	13 402 (19.1)	60.1 (15.73)	
**Education**			
Associate degree and less	25 696 (36.6)	59.94 (16.67)	F(2, 70,177): 73.395 *p*< 0.001^**^
Bachelor	26 373 (37.6)	59.09 (15.49)	
Master and higher	18 111 (25.8)	60.91 (14.51)	
**Economic situation**			
Good	11 449 (16.3)	63.26 (14.93)	t: 25.43, df: 70 177 *p*< 0.001^*^
Moderate and poor	58 730 (83.7)	59.21 (15.72)	

^*^Independent t-test

^**^One-way ANOVA

### Evidence for factorial structural validity

**Fig. 1:**
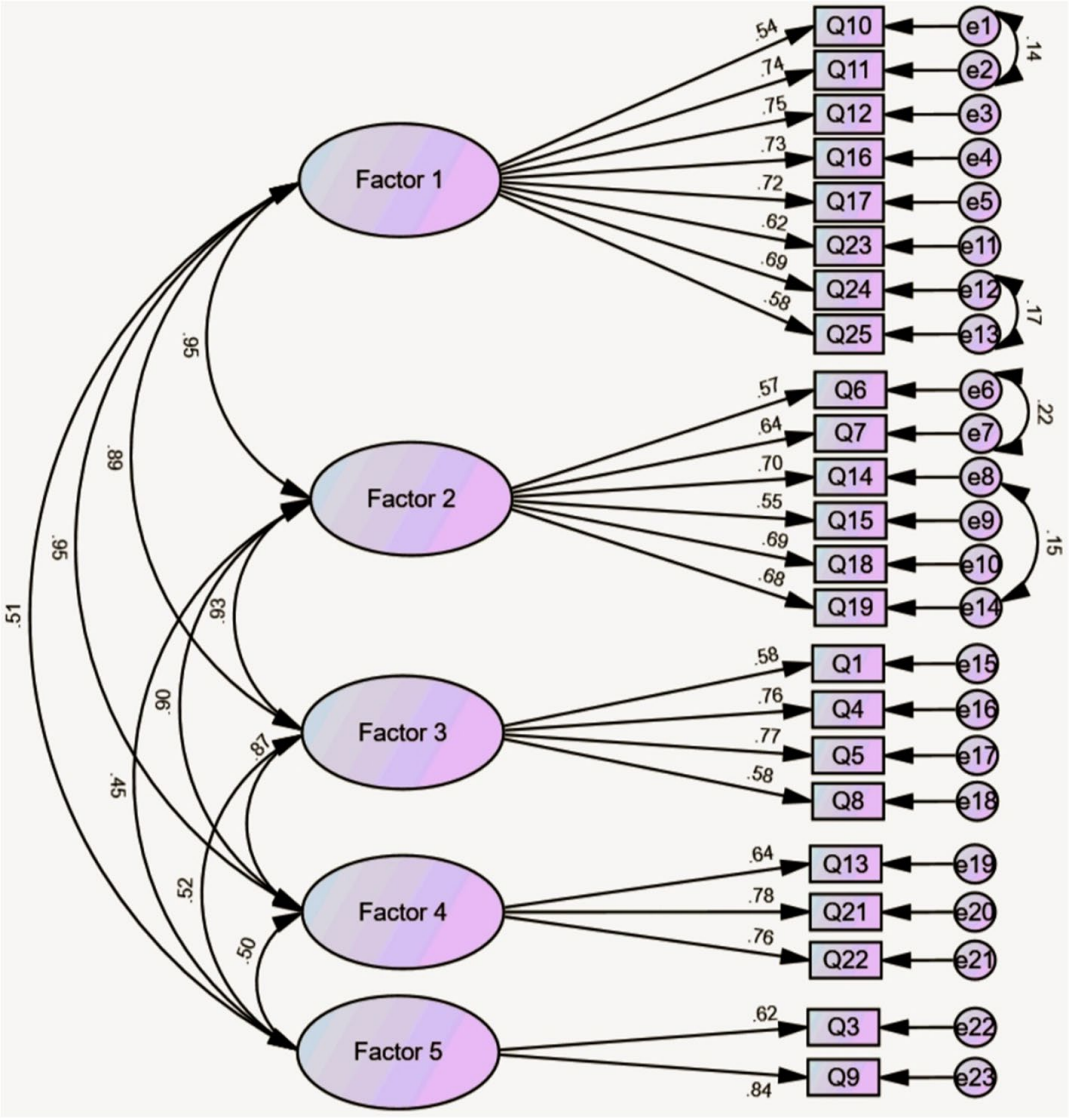
The results of the confirmatory factor analysis for first-order model (*n* = 70 108)

The maximum likelihood CFA was performed to validity and confirmed the psychometric properties of the original five-factor structure of CD-RISC using the Iranian population. Results of the several model fit indices showed that the five-factor model fits the data well as evidenced by an acceptable goodness-of-fit (CFI = 0.917, NFI = 0.916, TLI = 0.906, SRMR = 0.036, RMSEA (90% C.I.) = 0.059 [0.059, 0.059]). However, results showed that the factor loading for item 2 and item 20 were less than 0.5. Therefore, to enhance the psychometric qualities of the Iranian-CD-RISC, the original five-factor model was slightly modified by removing item 2 and item 20 and co-variating the error terms of three pairs of the items (between item 10 and item 11, item 24 and item 25, item 6 and item 7, item 14 and 19, and item 21 and item 22) following the modification indices (Fig. 1). The modified five-factor structure of the Iranian-CD-RISC demonstrated a good mode fit (CFI = 0.937, NFI = 0.937, TLI = 0.926, SRMR = 0.033, RMSEA (90% C.I.) = 0.057 [0.057, 0.058]).

### Construct reliability and validity

The results showed that the five-factor Iranian-CD-RISC has an acceptable internal consistency and construct reliability. As shown in Table 2, Cronbach’s alpha for Factor 1 (0.869), Factor 2 (0.811), Factor 3 (0.762), and factor 4 (0.761) were greater than 0.7, indicating good internal consistency. As for Factor 5, the Cronbach’s alpha (0.681) was slightly less than 0.7, it is still acceptable for psychological construct when the value is greater than 0.6 [23, 24], the reason of lower Cronbach’s alpha for Factor 3 could be due to fewer items of this construct. Moreover, the CR (Factor 1: 0.869; Factor 2: 0.804; Factor 3: 0.770; Factor 4: 0.773; Factor 5: 0.697) and MaxR (Factor 1: 0.878; Factor 2: 0.810; Factor 3: 0.793; Factor 4: 0.784; Factor 5: 0.747) for all five factors showed good construct reliability. As for convergent validity, only AVE for Factor 4 and Factor 5 were greater than 0.5. However, Factor 1 (0.458), Factor 2 (0.408), Factor 3 (0.460) were slightly less than 0.5, AVE is a strict measurement for convergent validity, using CR more than 0.7 alone can assess convergent validity in psychological studies [18, 24]. Therefore, following the results of the CR and MaxR, the convergent validity for all five factors was achieved. With respect to discriminant validity, the results of HTMT correlation analysis showed that the acceptabe discreminat validty between Factor 1 and Factor 3 (0.884), Factor 1 and Factor 5 (0.512), Factor 2 and Factor 5 (0.441), Fatcor 3 and Factor 4 (0.884), Factor 3 and Factor 5 (0.541), and Factor 4 and Factor 5 (0.525). However, the discriminant validity between Factor 1 and Factor 2 (0.926), Fatcor 1 and Factor 4 (0.956), Factor 2 and Factor 3 (0.932), and Factor 2 and Factor 4 (0.906) were not estabilihsed since there was a strong correlation between those factors in the Iranian-CD-RISC first-order model. Hence, the results suggested that there might be a second-order latent construct contained within these factors [25]. Therefore, we performed a second-order assessment to conform the Iranian-CD-RISC.

**Table 2:** The results of the internal consistency, construct reliability, and convergent validity

Factor	Cronbach’s alpha	CR	MaxR	AVE
Factor 1	.895	.869	.878	.458
Factor 2	.811	.804	.810	.408
Factor 3	.762	.770	.793	.460
Factor 4	.761	.773	.784	.534
Factor 5	.681	.697	.747	.541

### Second-order construct

**Fig. 2:**
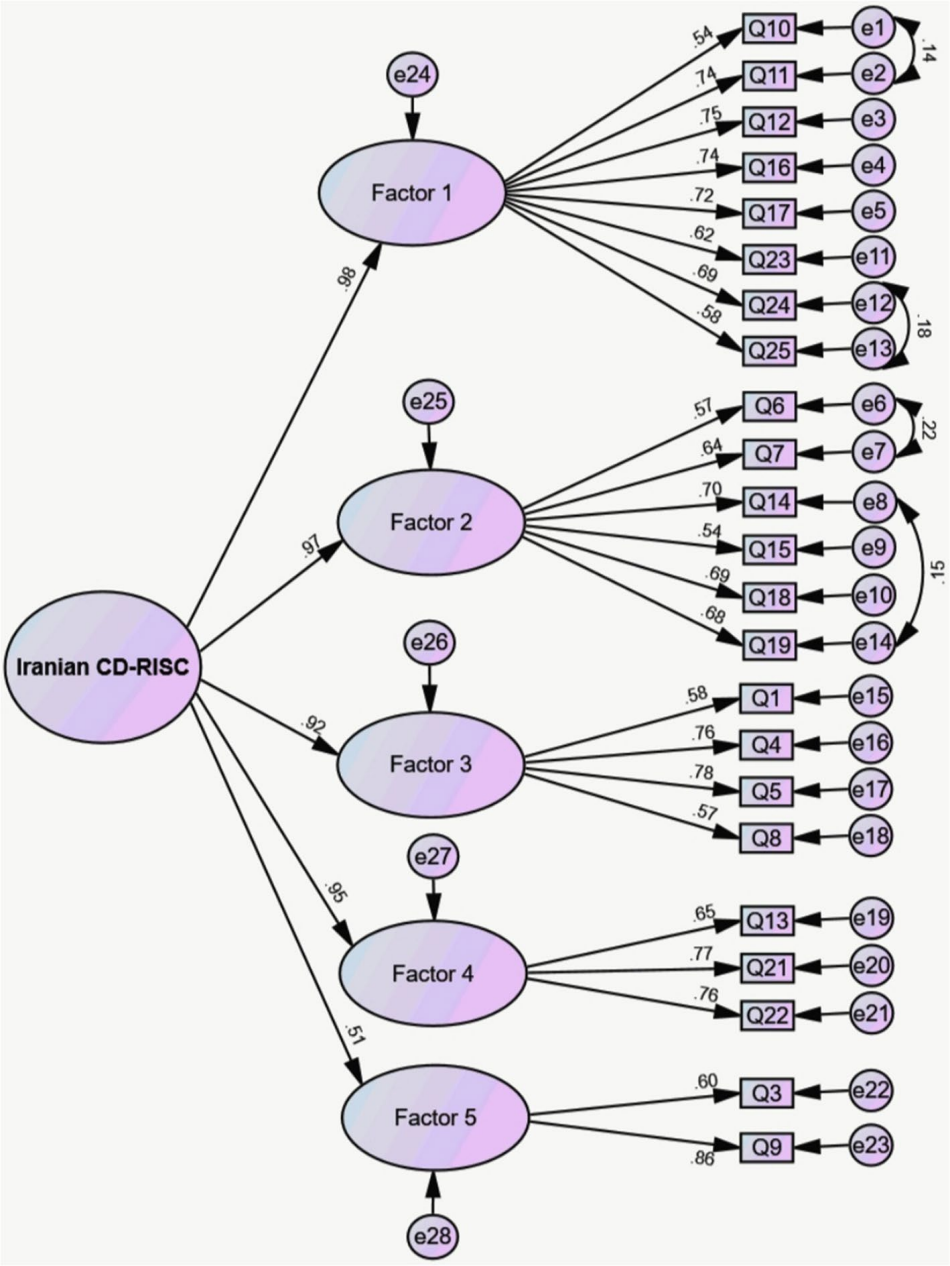
The results of the confirmatory factor analysis for second-order model (*n* = 70 108)

The results of the second-order latent construct assessment showed a strong goodness-of fit (CFI = 0.923, NFI = 0.923, TLI = 0.913, SRMR = 0.034, RMSEA (90% C.I.) = 0.057 [0.056, 0.057]). As shown in Fig. 2, factor loadings of each item of the first-order construct were greater than 0.5 and statistically significant. Moreover, the results showed that the CR (0.945) and MaxR (0.982) of the second-order construct more than 0.7, and AVE of the second-order construct was 0.782 which is more than the cult-off value of 0.5, indicating good construct reliability and validity of the second-order construct.

### Measurement invariance for gender

Analysis of measurement invariance for gender revealed both metric invariance (Δχ^2^ (22) = 194.62; *p* < 0.001, ΔCFI = 0.001 and ΔRMSEA = -0.001) and scalar invariance (Δχ^2^ (22) = 1943.82; *p* < 0.001, ΔCFI = -0.003 and ΔRMSEA = 0.001) according to the ΔCFI and ΔRMSEA criteria but not according to the Δχ^2^ criterium. However, it is well known that the χ^2^ statistic is inflated by large sample sizes as is the case in this study. This leads researchers to devalue this statistic when evaluating invariance (see, e.g.,[22]).

## Discussion

Resilience is a complex concept influenced by internal strengths and external capacities; thus, different definitions and constructs have been developed based on researchers’ perspectives and disciplines [26, 27]. Previous studies regarding the relationship between resilience and demographic characteristics have presented different results. Most of the studies on resilience have been conducted on specific populations and limited studies have been conducted on the general population. The results of different research have created two different paths regarding the relationship between age and resilience. Some researchers believe that with increasing age, resilience decreases due to the reduction of physical and mental strength, and alternatively, researchers believe that with increasing age and gaining more experience and strengthening adaptation strategies, increases resilience [28]. In the current research, in line with this second approach, resilience has increased with age. In term of the relationship between gender and resilience, researchers reported different results. Some studies reported higher levels of resilience score in women [29, 30] and others found higher resilience in men [31–34]. The result of this study is in line with the latter. The higher level of resilience in men may be related to the socio-cultural context of Iran in which men have greater opportunities and social interactions. In this study, married people had higher resilience than single people. This result is in line with results of some others studies [30, 32, 35], and may be because married people have better support networks than single people. On the other hand, researchers show that people with higher education had higher resilience scores [29, 30, 35, 36], the results of this confirmed this finding, too. In term of economic status, the result of this study showed that people with good economic status had higher level of resilience which is line of the Kocalevent et al. [33]; whose study had a large sample size. Further studies are need to analyze the relationship between resilience and educational level and economic status.

There are 15 questionnaires and scales available that assess resilience, according to a report of one systematic review. Some of these scales with high scores in terms of psychometric properties are [3] the Resilience Scale for Adults [37], the Brief Resilience Scale [38], and the CD-RISC. The resilience scale for adults was designed to examine protective factors (intrapersonal and interpersonal) that facilitate adaptation to psychosocial stresses. The Brief Resilience Scale was designed to assess the ability to recover from stress but the CD-RISC was designed to assess the ability to cope with stress [3].

Connor-Davidson’s resilience scale has been used in different languages and cultures and has been psychometrically evaluated in Iranian adolescents with cancer [14, 39]. However, adolescents and the general population are different in cognitive and language development [40]. Furthermore, their perception of stress and reactions would be different. Our study aimed to assess the psychometric properties of this scale in a large sample of the general population during COVID-19. The study results showed that the five-factor Iranian-Connor-Davidson Resilience scale has acceptable internal consistency, construct reliability, and convergent validity after removing items 2 and 20. Nevertheless, discriminant validity between factors was not established, as a strong correlation was found among the factors in the Iranian-CD-RISC first-order model. Therefore, to confirm the Iranian-CD-RISC, we performed a second-order assessment indicating good construct reliability and validity. A Chinese study on adolescents is consistent with ours in that the 5-factor structure model has better fit indices than the 3-factor model [41].

Whereas other studies have presented a four-factor [42–44], a three-factor [45, 46], and two-factor structures [47, 48] that is differ from the original scale and ours.

A variety of factors or items might be due to different contexts (culture and population) as they could influence the meaning and perception of the resilience of individuals [47]. Differences in personal characteristics and perception of the resilience concept or having diverse interpretations from scale items as well as using various strategies to deal with adversities may explain the differences in the models [41].

Based on some evidence, resilience is a dynamic concept that transforms during the development stages of the life course; therefore, variations in factors and items of the previous studies may be due to having different samples (adolescents) from ours (general population) [40]. Furthermore, the approach (orthogonal or oblique rotation method) may have been associated with obtaining different study results.

Our confirmed 5-factor model had more strong indices, including RMSEA (0.59), compared to the CFA results of the other studies in which RMSEA was obtained at less than 0.50 [41, 47].

This study was conducted in a large and non-clinical sample that facilitated performing CFA to propose a 5-factor model of CD-RISC. Under these conditions, we were able to evaluate the relationships between factors and individual characteristics during a pandemic. Therefore, our study results could help identify at-risk populations in Iran who need help and psychosocial support.

Analysis of measurement invariance indicated that the Farsi version of the Connor-Davidson Resilience Scale displayed strong scalar invariance; and it can thus be used to measure resilience in both men and women ensuring the validity and reliability of gender comparisons in adult Iranians.

This study has some limitations. One of these limitations is that data were gathered via online questionnaires, and we could not include individuals in the same quota from all parts of the country and obtain diverse demographic characteristics. Therefore, the generalizability of our data needs to be considered carefully. Nevertheless, our study has multiple strength: We had a very large sample size; also, the assessment of discriminant validity and measurement invariance for gender are other strengths of this study.

## Conclusions

Despite limitations mentioned, the present investigation shows that the 23-item version of CD-RISC is a reliable and valid scale to measure resilience as a complex construct in an Iranian population. This scale can be used in the general population for screening individuals for their resiliency. This predictive validity is of great importance for our study. Future research can reevaluate the discriminant validity of the scale.

## Data Availability

The datasets used and analyzed during the current study are available from the corresponding author on reasonable request.

## Abbreviations

CD-RISC: Connor-Davidson Resilience Scale
COVID-19: Coronavirus disease of 2019
URL: Uniform Resource Locator
ANOVA: One-Way Analysis of Variance
CFA: Confirmatory Factor Analysis
CFI: Comparative Fit Index
NFI: Normed Fit Index
TLI: Tucker–Lewis index
SRMR: Standardized Root Mean Square Residual
RMSEA: Root Mean Square Error of Approximation
CR: Composite Reliability
MaxR: Maximum Reliability
AVE: Average Variance Extracted
HTMT: Heterotrait-monotrait ratio of correlations
C.I: Confidence Interval
